# Supplementary material for: End Sequence Analysis Toolkit (ESAT) expands the extractable information from single-cell RNA-seq data
Source: Genome Res. 2016 Oct;26(10):1397–410. doi: 10.1101/gr.207902.116 (PMC5052061; doi:10.1101/gr.207902.116)
Supplement: Supplemental Material [file supp_gr.207902.116_Supp_Materials.tar.gz › ESAT-esat_v0.1/doc/html/classbroad_1_1core_1_1siphy_1_1_conserved_p_w_m_scanner-members.html]

ESAT: Member List


ESAT


- Main Page
- Related Pages
- Packages
- Classes
- Files

- Class List
- Class Index
- Class Hierarchy
- Class Members

All Classes Namespaces Files Functions Variables Enumerator Pages

- broad
- core
- siphy
- ConservedPWMScanner

broad.core.siphy.ConservedPWMScanner Member List

This is the complete list of members for broad.core.siphy.ConservedPWMScanner, including all inherited members.

|  |  |  |
| --- | --- | --- |
| ConservedPWMScanner(String pwmFile) | broad.core.siphy.ConservedPWMScanner |  |
| ConservedPWMScanner(List< PositionWeightMatrix > matrices) | broad.core.siphy.ConservedPWMScanner |  |
| getAlignmentChunkSize() | broad.core.siphy.ConservedPWMScanner |  |
| getPWMiterator() | broad.core.siphy.ConservedPWMScanner |  |
| scan(MAFIO chrMafIO, List< BED > annotations, int shuffles, float seedMinScore) | broad.core.siphy.ConservedPWMScanner |  |
| setAlignmentChunkSize(int alignmentChunkSize) | broad.core.siphy.ConservedPWMScanner |  |
| setBackgroundModel(AnnotationReader<?extends GenomicAnnotation > reader, Map< String, Sequence > org) | broad.core.siphy.ConservedPWMScanner |  |
| setIgnoreList(List< String > ignoreList) | broad.core.siphy.ConservedPWMScanner |  |
| setModel(EvolutionaryModel model) | broad.core.siphy.ConservedPWMScanner |  |
| slidePWM(PositionWeightMatrix pwm, double minSeedScore, short[] encodedRef, List< int[]> ungappedChunks, int numPermutations) | broad.core.siphy.ConservedPWMScanner | protected |


---

Generated on Fri Aug 15 2014 15:10:56 for ESAT by  

 1.8.7
